# Osteoporosis in ankylosing spondylitis - prevalence, risk factors and methods of assessment

**DOI:** 10.1186/ar3833

**Published:** 2012-05-08

**Authors:** Eva Klingberg, Mattias Lorentzon, Dan Mellström, Mats Geijer, Jan Göthlin, Elisabet Hilme, Martin Hedberg, Hans Carlsten, Helena Forsblad-d'Elia

**Affiliations:** 1 Department of Rheumatology and Inflammation Research, Sahlgrenska Academy at University of Gothenburg, Guldhedsgatan 10A, S-413 46 Göteborg, Sweden; 2 Centre for Bone and Arthritis Research, Institute of Medicine, Sahlgrenska Academy at University of Gothenburg, Sahlgrenska University Hospital, Vita stråket 11, S-413 45 Göteborg, Sweden; 3 Centre for Medical Imaging and Physiology, Skåne University Hospital, Lund University, Getingevägen 4, S-221 85 Lund, Sweden; 4 Department of Radiology, Sahlgrenska University Hospital/Mölndal, Göteborgsvägen 31, S-431 30 Mölndal, Sweden; 5 Department of Rheumatology, Alingsås Lasarett, Södra Ringgatan 30, S-441 83 Alingsås, Sweden; 6 Department of Rheumatology, SÄS Borås, Brämhultsvägen 53, S-501 82 Borås, Sweden

## Abstract

**Introduction:**

Osteoporosis can be a complication of ankylosing spondylitis (AS), but diagnosing spinal osteoporosis can be difficult since pathologic new bone formation interferes with the assessment of the bone mineral density (BMD). The aims of the current study were to investigate prevalence and risk factors for reduced BMD in a Swedish cohort of AS patients, and to examine how progressive ankylosis influences BMD with the use of dual-energy x-ray absorptiometry (DXA) of the lumbar spine in different projections.

**Methods:**

Methods of assessment were questionnaires, back mobility tests, blood samples, lateral spine radiographs for syndesmophyte grading (mSASSS), DXA of the hip, radius and lumbar spine in anteroposterior (AP) and lateral projections with estimation of volumetric BMD (vBMD).

**Results:**

AS patients (modified New York criteria), 87 women and 117 men, mean age 50 ± 13 years and disease duration 15 ± 11 years were included. According to World Health Organization (WHO) criteria 21% osteoporosis and 44% osteopenia was diagnosed in patients > = 50 years. Under age 50 BMD below expected range for age was found in 5%. Interestingly lateral lumbar DXA showed significantly lower BMD and revealed significantly more cases with osteoporosis as compared with AP DXA. Lumbar vBMD was not different between sexes, but women had significantly more lumbar osteoporosis measured with AP DXA (*P *< 0.001). Men had significantly higher mSASSS (*P *< 0.001). Low BMD was associated with high age, disease duration, mSASSS, Bath Ankylosing Spondylitis Metrology Index (BASMI), inflammatory parameters and low body mass index (BMI). Increasing mSASSS correlated significantly with decreasing lateral and volumetric lumbar BMD, while AP lumbar BMD showed tendency to increase.

**Conclusions:**

Osteoporosis and osteopenia is common in AS and associated with high disease burden. Lateral and volumetric lumbar DXA are more sensitive than AP DXA in detecting osteoporosis and are less affected by syndesmophyte formation.

## Introduction

Ankylosing spondylitis (AS) is a chronic inflammatory rheumatic disease, mainly affecting the sacroiliacal joints, vertebrae and intervertebral discs, leading to syndesmophyte formation and impaired back mobility. In AS two enhanced but opposite bone remodelling processes are taking place in close vicinity within the spine; these are pathologic new bone formation in the cortical zone of the vertebrae, the zygapophyseal joints, and the ligamentous apparatus and excessive loss of trabecular bone in the centre of the vertebral body leading to osteoporosis. Earlier studies have demonstrated an increased prevalence of osteoporosis and significantly lower bone mineral density (BMD) in AS patients compared with sex and age matched controls [1,2]. Osteoporosis has also been shown to be present in mild AS and in early disease [2,3]. AS patients have a high risk of vertebral fractures, which can be complicated by neurological injuries [4,5].

In advanced AS it can be difficult to interpret lumbar spine BMD measured with dual-energy x-ray absorptiometry (DXA) in the anteroposterior (AP) projection. The new bone formation that is characteristic of AS causes an overestimation of the total BMD and values can be normal or high, even when osteoporosis is present. Spinal hyperostosis in AS is frequently located around the zygapophyseal joints, the vertebral endplates and the annulus fibrosus of the discs, and to a lesser extent, on the lateral sides [6]. Lateral DXA scanning of the lumbar spine allows exclusive examination of the vertebral body, which consists of 80% trabecular bone, and excludes the zygapophyseal joints, endplates and both anterior and posterior syndesmophytes from the measurement. Consequently, lateral scanning could be a way to reduce the problem of overestimation of lumbar spine BMD in AS. With older single-beam DXA systems patients were examined lying on their side and the precision was low. Modern lateral scans with the patient positioned supine offer precision similar to regular AP spine DXA scans [7].

The combination of AP and lateral DXA allows assessment of three-dimensional volumetric BMD (vBMD = bone mineral content/volume). Areal BMD (aBMD) is measured two-dimensionally, without taking the size of bone in the third dimension into account. Consequently, aBMD depends on both bone density and bone size, whereas vBMD is independent of bone size. Men have larger bones than women and consequently have higher aBMD, but vBMD is equal in both sexes [8,9].

BMD varies between different populations. Sweden has a high prevalence of osteoporosis and osteoporosis-related fractures [10-12]. Although studies of osteoporosis in AS have been performed in other countries, this is to our knowledge the first study in Scandinavia. The aims of the present study were to investigate the prevalence of, and risk factors for osteoporosis in patients with AS, to compare lumbar spine BMD measured in the AP and lateral projection, including estimated vBMD, and to study how these measures change with progressive ankylosis.

## Materials and methods

### Patients

The patients were recruited from three participating centres in western Sweden; the Rheumatology Clinic at Sahlgrenska University Hospital in Gothenburg and the Rheumatology Clinics at the Borås and Alingsås county hospitals. The inclusion criterion was AS according to the modified New York criteria [13]. Exclusion criteria were psoriasis, inflammatory bowel disease, dementia, pregnancy and difficulties in understanding Swedish. All patients meeting the study criteria were invited to participate. Informed consent was obtained according to the Declaration of Helsinki. The study was approved by the regional ethics committee in Gothenburg.

### Physical examination and questionnaires

Physical examinations, including the Bath Ankylosing Spondylitis Metrology Index (BASMI), were all performed by the same physician (EK). The patients answered questionnaires concerning risk factors for osteoporosis, medical history, medication and the Bath Ankylosing Spondylitis Disease Activity Index (BASDAI), Bath Ankylosing Spondylitis Functional Index (BASFI) and Bath Ankylosing Spondylitis Patient Global score (BAS-G) [14-17]. The Ankylosing Spondylitis Disease Activity Score (ASDAS) was calculated using a previously described formula [18,19].

Physical activity was divided into three levels of intensity (light, moderate and heavy) and reported in hours per week for leisure time, at home and at work, using two validated questionnaires; the Leisure Time Physical Activity Instrument (LTPAI) and Physical Activity at Home and Work Instrument (PAHWI) [20]. Lifetime use of glucocorticoids, converted into milligrams of prednisone, was estimated by examining the medical records and questioning patients about previous glucocorticoid injections and oral prednisone use. Fracture risk assessment (FRAX) for the % ten-year probability of major osteoporotic and vertebral fractures respectively, was calculated using the FRAX tool developed from a Swedish cohort. The FRAX tool applies only to patients 40 years or older. The probability of fracture is calculated using known risk factors for fractures, and BMD of the femoral neck [21].

### Bone mineral density

BMD of the lumbar spine in the AP (vertebrae L1 to L4) and lateral (L2 to L4) projections, the left hip (femoral neck and total hip regions) and the non-dominant forearm (radius) was measured using a DXA scanner (Hologic Discovery A, Hologic Inc, Bedford, MA, USA). The coefficients of variation for repeat scan precision were 0.39% for the AP and 0.60% for the lateral lumbar DXA measurements. For patients aged 50 years or older the following World Health Organization (WHO) definitions of osteopenia and osteoporosis were used: osteopenia, T-score < -1 to > -2.5 SD (compared to the young normal mean), and osteoporosis, T-score ≤ -2.5 SD. The lowest value of BMD measured in the lumbar spine, total hip or femoral neck was used [22]. For patients under the age of 50 a Z-score ≤ -2.0 SD (compared to the age-matched mean) was considered to be below the expected range for age [23]. For calculation of T- and Z-scores the BMD values of the patients were compared with reference values provided by the DXA scanner analysis software. T- and Z-scores were not available for lateral lumbar spine DXA measured in men or for lumbar spine vBMD measured in men or women.

### Radiography

Lateral radiographs of the cervical, thoracic and lumbar spine were acquired and changes related to AS were assessed using the modified Stoke Ankylosing Spondylitis Spine Score (mSASSS). The score includes the anterior corners of vertebrae C2 to T1 and T12 to S1, which are graded with 0 to 3 points each (0 = normal, 1 = erosion, sclerosis or squaring, 2 = syndesmophyte, 3 = bridging syndesmophyte). The remaining thoracic spine is not included in the score. The scoring scale ranges from 0 to 72 [24].

### Laboratory tests

Blood samples were analysed by standard laboratory techniques at the participating hospitals. The mean level of erythrocyte sedimentation rate (ESR) during the last five years was calculated using the first recorded ESR test for each year. When the patient had an infection the concomitant ESR test was excluded and replaced by the subsequent test.

### Statistics

Statistical analyses were performed using PASW Statistics 18.0 (SPSS Inc., IBM, Chicago USA). Descriptive statistics are presented as median and range and/or mean and standard deviation (SD). The *t-*test was used for comparison of normally distributed demographic and disease-related variables and the Mann-Whitney U-test was used to analyse variables that were not normally distributed. The chi-square test was used to compare categorical variables. Correlations were calculated using Spearman's correlation (r_s_). For dichotomous variables, yes was coded 1 and no was coded 0. All tests were two-tailed and *P *< 0.05 was considered statistically significant. Linear regression was run with BMD at different measurement sites as the outcome and logistic regression was run with the categorical variable low BMD T-score (yes/no) as the outcome. Covariates in both calculations were the variables that were significantly correlated with BMD in the first analyses.

## Results

Of the 538 AS patients registered in the hospitals' databases, 177 patients did not meet the modified New York criteria for AS or had exclusion criteria. Out of a total of 361 patients invited to participate, 72 declined, 60 did not respond to the invitation and 19 did not meet the inclusion criteria. Of the 210 patients included, 6 did not attend the DXA or radiography appointments and were therefore excluded. The patients who fulfilled the inclusion criteria but declined to participate or did not respond to the invitation (*n *= 151) were significantly younger than the patients included in the study (46 ± 13 years vs. 50 ± 13 years; *P *= 0.007), but the sex distribution was the same among patients who were or were not included.

A total of 204 patients completed the study; 87 (43%) women and 117 (57%) men. The mean age (50 ± 13 years), time since onset of AS symptoms (24 ± 13 years) and time since diagnosis (15 ± 11 years) were evenly distributed between the sexes. Demographic and disease-related variables are shown in Table 1.

**Table 1:** Characteristics of 204 patients with ankylosing spondylitis in western Sweden

		**Patients**, number (%)	Median (range)	Mean ± SD
**Demographic variables**				
Sex	Women	87 (43)		
	Men	117 (57)		
Age, years		NA	49 (17 to 78)	50 ± 13
Postmenopausal women		45/87 (52)		
Heredity for fractures		57 (28)		
History of vertebral fracture		3 (1)		
History of non-vertebral fracture		18 (9)		
Current smokers		24 (12)		
Ever smoked > 6 months		101 (50)		
Daily calcium intake from dairy products		NA	600 (0 to 2,640)	668 ± 397
BMI, kg/m^2^			25 (19 to 46)	26 ± 4
LTPAI total, hours			6 (0 to 42)	7 ± 6
PAHWI total, hours			45 (0 to 160)	40 ± 21
FRAX major osteoporotic fracture (%) (patients ≥ 40 years)			6.7 (1.2 to 68.0)	9.9 ± 9.7
FRAX hip fracture (%) (patients ≥ 40 years)			0.8 (0 to 56.0)	2.4 ± 6.0
**Disease-related variables**				
Years since onset of symptoms			24 (2 to 55)	24 ± 13
Years since diagnosis			12 (1 to 47)	15 ± 11
History of anterior uveitis		102 (50)		
History of peripheral arthritis		120 (59)		
History of coxitis		17 (8)		
BASMI, score			3.0 (0.6 to 7.4)	3.1 ± 1.6
BASDAI, score			3.5 (0.0 to 9.6)	3.6 ± 2.1
BASFI, score			2.3 (0.0 to 8.7)	2.7 ± 2.1
BAS-G1, score (last week)			2.9 (0.0 to 10.0)	3.4 ± 2.6
BAS-G2, score (last 6 months)			3.4 (0.0 to 9.7)	3.8 ± 2.6
ASDAS, score			2.3 (0.8 to 5.9)	2.4 ± 0.9
mSASSS, score			5.5 (0.0 to 72.0)	14.2 ± 19.2
Mean ESR, mm/h (2004 to 2008)			16 (2 to 102)	19 ± 15
ESR, mm/h (at inclusion 2009)			11 (2 to 105)	15 ± 14
CRP, mg/L			5 (3 to 80)	9 ± 10
Hemoglobin, g/L			139 (105 to 166)	139 ± 13
WBC, × 10^9^/L			6.7 (2.7 to 18.1)	7.0 ± 2.1
PLT, × 10^9^/L			287 (133 to 506)	299 ± 75
Creatinine, μmol/L			70 (43 to 148)	71 ± 15
HLA-B27 positive		178 (87)		
Patients on NSAID		158 (77)		
Patients on DMARD		62 (30)		
Patients on TNF inhibitor		42 (21)		
Patients on GC		7 (3)		
GC lifetime use, mg prednisone		NA	100 (0, 56390)	1397 ± 5775
Patients on bisphosphonates		8 (4)		
Patients on HRT		5 (2)		
Patients on calcium and vitamin D		24 (12)		
**Bone mineral density**				
aBMD AP lumbar spine, g/cm^2^			1.02 (0.63 to 1.54)	1.02 ± 0.17
aBMD lateral lumbar spine, g/cm^2^			0.72 (0.32 to 1.13)	0.73 ± 0.14
vBMD lumbar spine, g/cm^3^			0.19 (0.09 to 0.27)	0.19 ± 0.03
BMD total hip, g/cm^2^			0.93 (0.54 to 1.42)	0.94 ± 0.14
BMD femoral neck, g/cm^2^			0.78 (0.48 to 1.20)	0.78 ± 0.13
BMD radius total, g/cm^2^			0.61 (0.40 to 0.78)	0.61 ± 0.08

BMI, body mass index; LTPAI, Leisure Time Physical Activity Instrument; PAHWI, Physical Activity at Home and Work Instrument; FRAX, World Health Organization Fracture Risk Assessment Tool (% 10 year probability of major osteoporotic, and vertebral fracture respectively, corresponding to patients aged 40 years or older); BASMI, Bath Ankylosing Spondylitis Metrology Index; BASDAI, Bath Ankylosing Spondylitis Disease Activity Index; BASFI, Bath Ankylosing Spondylitis Functional Index; BAS-G1 and G2, Bath Ankylosing Spondylitis Patient Global Score; ASDAS, ankylosing spondylitis disease activity score; mSASSS, modified Stoke Ankylosing Spondylitis Spine Score; ESR, erythrocyte sedimentation rate; CRP, C-reactive protein; PLT, platelet count; WBC, white blood cell count; NSAID, non-steroidal anti-inflammatory drug; DMARD, disease modifying anti-rheumatic drug; TNF, tumour necrosis factor; GC, glucocorticoid; HRT, hormone replacement therapy; aBMD, areal bone mineral density; AP, anteroposterior; vBMD, volumetric BMD

### Prevalence of reduced BMD

Among patients under 50 years of age (*n *= 103), 35 patients (34%) had a BMD Z-score < -1.0 at the hip and/or AP lumbar spine and 5 patients (4.9%) had BMD below the expected range for age, that is, a Z-score ≤ -2.0. Among patients 50 years of age or older (*n *= 101), 44 (43.6%) had osteopenia and 21 (20.8%) had osteoporosis using the WHO definition [22]. The lumbar spine was the most common location for osteoporosis or BMD below the expected range for age (10%), followed by radius (8%) and femoral neck (5%). The prevalence of reduced BMD in different locations is shown in Table 2.

**Table 2:** Prevalence of reduced BMD measured by dual-energy x-ray absorptiometry at different skeletal sites.

Measurement site	**Age-group**, years	**BMD**, mean ± SD	**T-score**, mean ± SD	**Z-score**, mean ± SD	Patients with osteoporosis/BMD below expected range for age, number (%)^a^	**Patients with osteopenia**, number (%)	Patients with normal BMD number (%)
**Women**							
AP lumbar spine	< 50	1.014 ± 0.134	-0.30 ± 1.22	0.02 ± 1.25	2 (5)	0 (0)	41 (95)
	≥ 50	0.899 ± 0.150	-1.35 ± 1.37	0.14 ± 1.38	12 (27)	15 (34)	17 (39)
Lat lumbar spine	< 50	0.741 ± 0.102	-0.94 ± 1.22	-0.25 ± 1.26	3 (7)	0 (0)	40 (93)
	≥ 50	0.616 ± 0.114	-2.43 ± 1.36	-0.05 ± 1.42	20 (45)	15 (34)	9 (20)
Total hip	< 50	0.925 ± 0.122	-0.13 ± 1.00	0.07 ± 0.99	0 (0)	0 (0)	43 (100)
	≥ 50	0.832 ± 0.108	-0.89 ± 0.88	0.10 ± 0.85	0 (0)	20 (46)	23 (54)
Femoral neck	< 50	0.804 ± 0.127	-0.41 ± 1.15	-0.06 ± 1.13	0(0)	0 (0)	43 (100)
	≥ 50	0.702 ± 0.103	-1.32 ± 0.93	-0.01 ± 0.91	4 (9)	24 (56)	15 (35)
Total radius	< 50	0.576 ± 0.038	-0.05 ± 0.70	0.32 ± 0.74	0(0)	0 (0)	43 (100)
	≥ 50	0.517 ± 0.065	-1.14 ± 1.21	0.18 ± 1.09	7 (16)	17 (39)	20 (45)
**Men**							
AP lumbar spine	< 50	1.057 ± 0.154	-0.31 ± 1.41	-0.18 ± 1.41	3 (5)	0 (0)	57 (95)
	≥ 50	1.092 ± 0.184	0.18 ± 1.67	0.63 ± 1.72	4 (7)	8 (14)	44 (79)
Lat lumbar spine	< 50	0.782 ± 0.131			NA	NA	NA
	≥ 50	0.740 ± 0.132			NA	NA	NA
Total hip	< 50	0.999 ± 0.137	-0.23 ± 0.92	-0.05 ± 0.90	1 (2)	0 (0)	59 (98)
	≥ 50	0.963 ± 0.136	-0.46 ± 0.90	0.00 ± 0.93	0 (0)	19 (33)	38 (67)
Femoral neck	< 50	0.842 ± 0.130	-0.67 ± 0.95	-0.21 ± 0.93	1 (2)	0 (0)	59 (98)
	≥ 50	0.773 ± 0.122	-1.16 ± 0.90	-0.21 ± 0.90	5 (9)	25 (44)	27 (47)
Total radius	< 50	0.668 ± 0.050	-0.34 ± 0.93	-0.12 ± 0.91	1 (2)	0 (0)	59 (98)
	≥ 50	0.633 ± 0.063	-1.03 ± 1.21	-0.25 ± 1.20	9 (16)	18 (33)	28 (51)

Definition of reduced BMD in patients younger than 50 years, BMD below expected range for age (Z-score ≤ -2.0 SD), BMD within expected range for age (Z-score > -2.0 SD); in patients 50 years or older, World Health Organization definition of osteoporosis: T-score ≤ - 2.5 SD, osteopenia: T-score < -1 to > -2.5 SD; normal BMD, T-score ≥ -1SD. For lateral lumbar spine BMD in men there are no reference values available for calculation of T- or Z-scores. ^a^Data presented as the sum of patients younger than 50 years, with a Z-score ≤ -2.0 SD and patients 50 years or older with a T-score ≤ -2.5 SD.

AP, anteroposterior; Lat, lateral; BMD, bone mineral density.

**Figure 1:**
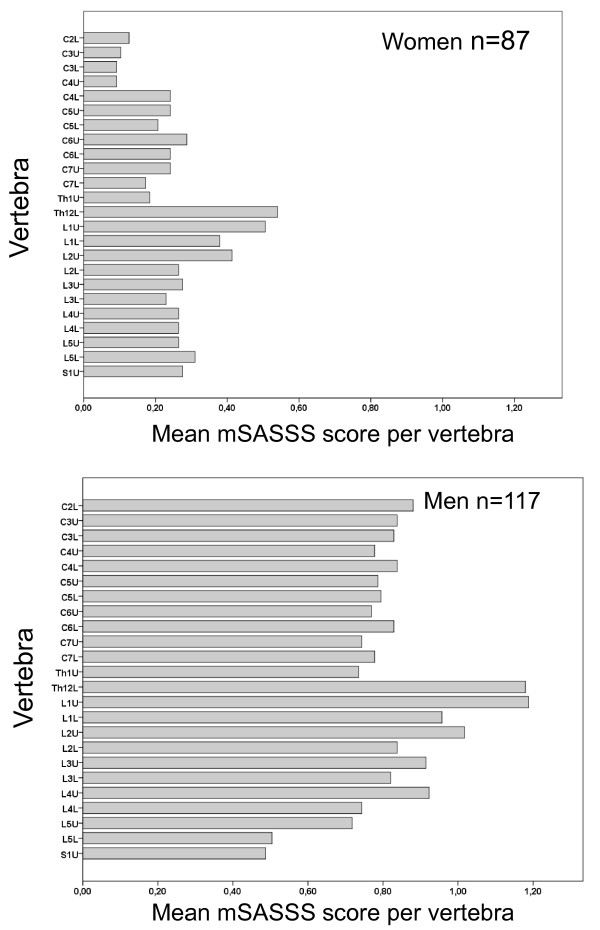
**Mean modified Stoke Ankylosing Spondylitis Spine Score (mSASSS) per vertebra in women and men**. C, cervical vertebra; Th, thoracic vertebra; L, lumbar vertebra; S, sacral vertebra; the upper and lower anterior corners of the vertebrae are denoted by U and L, respectively.

Men had significantly lower BMD at the femoral neck (mean Z-score -0.215, *P *= 0.012), compared with the age- and sex-matched reference values in the DXA-scanner software. Mean Z-scores were negative at all measurement sites except the lumbar spine (AP projection), but was not significantly different to zero. Significantly more women aged 50 years or older had osteoporosis on measurement of AP lumbar spine than men (12/44, 30% vs. 4/56, 7%, *P *< 0.001). Consequently, diagnosis of osteoporosis and osteopenia using the WHO definition was more prevalent among women (13/44, 30% and 21/44, 48%, respectively) compared with men (8/56, 14% and 23/56, 41%, respectively; *P *= 0.042). BMD below the expected range for age was equally common in women and men below 50. Men had a significantly higher mSASSS score (median 8, range 0 to 72) than women (median 2, range 0 to 46) (*P *< 0.001) (Figure 1). Men had significantly higher aBMD at all measurement sites (*P *< 0.001 for every location), but for lumbar vBMD there was no significant difference between the sexes (*P *= 0.36). The BASDAI, BASFI, BASMI and ASDAS were evenly distributed among women and men.

A total of 36% of women and 8% of men had ever undergone a DXA examination before the study. Eight patients were on bisphosphonates at the inclusion. After the study new treatment with calcium, vitamin D and bisphosphonates, according to national guidelines, was initiated in 30 patients (14.2%) and calcium and vitamin D alone was initiated in 28 patients (13.2%).

### Risk factors for reduced BMD

The demographic and disease-related variables explored for association with BMD are shown in Table 1. Low BMD correlated significantly with demographic variables (female sex, older age, low body mass index (BMI), heredity for fractures, PAHWI and menopausal status for women) as well as disease-related variables (long disease duration, high BASMI, high mSASSS, history of coxitis, high lifetime use of prednisone, low hemoglobin and elevated inflammatory parameters, high mean ESR, C-reactive protein (CRP), white blood cell count (WBC), platelet count (PLT) (Table 3). No significant association was found between BMD and demographic variables (heredity for osteoporosis, smoking, alcohol intake or LTPAI) or disease-related variables (anterior uveitis, arthritis, BASDAI, ASDAS, BASFI or BAS-G). The (MHC class 1) HLAB27-negative patients had significantly lower BMD at the total radius compared with HLAB27-positive patients (0.564 ± 0.077 vs. 0.612 ± 0.779 g/cm^2^; *P *= 0.005), but there was no difference in BMD at other measurement sites and no difference in Z-scores for total radial BMD between HLAB27-negative and -positive patients. The HLAB27-negative patients were significantly more often female (*P *= 0.012).

**Table 3:** Demographic and disease-related variables showing significant correlation with BMD at different measurement sites.

Variables	AP lumbar aBMD	Lateral lumbar aBMD	Lumbar vBMD	Hip total aBMD	Femoral neck aBMD	Radius total aBMD
**Demographic**						
Sex	0.337 *P *< 0.001	0.294 *P *< 0.001	n.s.	0.380 *P *< 0.001	0.228 *P *= 0.001	0.677 *P *< 0.001
Age	n.s.	-0.282 *P *< 0.001	-0.413 *P *< 0.001	-0.222 *P *= 0.001	-0.322 *P *< 0.001	-0.328 *P *< 0.001
Menopause	-0.394 *P *< 0.001	-0.385 *P *< 0.001	-0.190 *P *= 0.007	-0.378 *P *< 0.001	-0.322 *P *< 0.001	-0.570 *P *< 0.001
Heredity for fractures	n.s.	n.s.	n.s.	-0.249 *P *< 0.001	-0.276 *P *< 0.001	-0.148 *P *= 0.035
BMI	0.273 *P *< 0.001	n.s.	n.s.	0.342 *P *< 0.001	0.255 *P *< 0.001	0.177 *P *= 0.012
PAHWI total	n.s.	0.179 *P *= 0.011	0.253 *P *< 0.001	n.s.	n.s.	0.178 *P *= 0.012
**Disease-related** **variables**						
Years since symptom debut	-0.180 *P *= 0.011	-0.335 *P *< 0.001	-0.429 *P *< 0.001	-0.315 *P *< 0.001	-0.376 *P *< 0.001	-0.270 *P *< 0.001
Years since diagnosis	-0.146 *P *= 0.039	-0.286 *P *< 0.001	-0.396 *P *< 0.001	-0.221 *P *= 0.002	-0.319 *P *< 0.001	n.s.
BASMI	n.s.	-0.260 *P *< 0.001	-0.405 *P *< 0.001	-0.257 *P *< 0.001	-0.379 *P *< 0.001	-0.195 *P *= 0.006
mSASSS	n.s.	n.s.	-0.386 *P *< 0.001	n.s.	-0.187 *P *= 0.008	n.s.
Coxitis, yes/no	-0.158 *P *= 0.025	-0.143 *P *= 0.042	n.s.	-0.189 *P *= 0.007	-0.161 *P *= 0.022	n.s.
Mean ESR 2004 to 2008	-0.172 *P *= 0.015	n.s.	n.s.	-0.168 *P *= 0.017	-0.155 *P *= 0.028	-0.222 *P *= 0.002
Lifetime prednisone use	n.s.	-0.241 *P *= 0.001	-0.236 *P *= 0.001	-0.222 *P *= 0.001	-0.269 *P *< 0.001	-0.316 *P *< 0.001
ESR	-0.162 *P *= 0.021	-0.221 *P *= 0.002	n.s.	-0.170 *P *= 0.016	n.s.	-0.285 *P *< 0.001
CRP	n.s.	n.s.	-0.163 *P *= 0.021	n.s.	n.s.	n.s.
Hemoglobin	0.261 *P *< 0.001	0.225 *P *= 0.001	n.s.	0.285 *P *< 0.001	0.179 *P *< 0.011	0.466 *P *< 0.001
WBC	-0.159 *P *= 0.024	-0.171 *P *= 0.015	n.s.	-0.146 *P *= 0.038	n.s.	n.s.
PLT	-0.197 *P *= 0.005	-0.155 *P *= 0.028	n.s.	n.s.	n.s.	n.s.

Results are presented as Spearman's correlation coefficients and *P*-values. Coding for categorical variables, 1 = yes, 0 = no; for sex, 1 = woman, 2 = man.

BMI, body mass index; PAHWI, Physical Activity at Home and Work Instrument; BASMI, Bath Ankylosing Spondylitis Metrology Index; mSASSS, modified Stoke Ankylosing Spondylitis Spine Score; ESR, erythrocyte sedimentation rate; CRP, C-reactive protein; WBC, white blood cell count; PLT, platelet count; AP, anteroposterior; aBMD, areal bone mineral density; vBMD, volumetric BMD; n.s., not significant.

The variables significantly correlated with BMD were entered as covariates in stepwise multiple linear regression analyses with BMD at different measurement sites as the outcome (Table 4). The most important covariates for low BMD were long disease duration, high BASMI, low BMI and menopause in women. AP lumbar spine BMD was mainly associated with demographic risk factors, whereas lateral lumbar spine BMD and vBMD were associated to a greater extent with disease-related risk factors.

**Table 4:** Results from multiple linear regression analyses with BMD at different measuring sites as the outcome.

	AP lumbar aBMD		Lateral lumbar aBMD		Lumbar vBMD		Total hip aBMD		Femoral neck aBMD		Total radius aBMD	
Constant all variables	0.829		0.539		0.2390		0.783		0.654		0.625	
**R^2 ^**all variables	0.290		0.245		0.266		0.402		0.343		0.591	
**R^2 ^**for demographic variables	0.254		0.163		0.171		0.338		0.279		0.569	
**R^2 ^**for disease related variables	0.079		0.181		0.276		0.174		0.198		0.253	

**Demographic variables**	**B**	*P*-value	**B**	*P*-value	**B**	*P*-value	**B**	*P*-value	**B**	*P*-value	**B**	*P*-value

Sex	NA		NA		NA		-0.056	0.009	NA		-0.082	< 0.001
Age, years	NA		NA		NA		NA		NA		-0.001	0.002
Menopause	-0.156	< 0.001	-0.104	< 0.001	NA		-0.053	0.048	-0.076	< 0.001	-0.035	0.009
BMI, kg/m^2^	0.014	< 0.001	NA		NA		0.014	< 0.001	0.011	< 0.001	0.004	< 0.001

**Disease-related variables**	**B**	*P *-value	**B**	*P *-value	**B**	*P *-value	**B**	*P *-value	**B**	*P *-value	**B**	*P *-value

Years since diagnosis	NA		-0.003	< 0.001	NA		NA		NA		NA	
Years since symptom debut	NA		NA		-0.001	< 0.001	-0.002	0.019	-0.002	0.028	NA	
BASMI	NA		NA		-0.006	< 0.001	-0.023	< 0.001	-0.029	< 0.001	-0.008	0.016
Hemoglobin	NA		0.002	0.008	NA		NA		NA		NA	
WBC	NA		NA		NA		-0.007	0.05	NA		NA	
PLT	0.000	0.003	NA		NA		NA		NA		NA	

All demographic and disease-related variables that were significantly correlated with BMD in the first analyses were entered as covariates. The table shows only covariates that remained significantly associated with BMD in the stepwise multiple linear regression models. Coding for categorical variables, 1 = yes, 0 = no; for sex, 1 = woman, 2 = man. Beta values are unstandardized regression coefficients.

AP, anteroposterior; BASMI, Bath Ankylosing Spondylitis Metrology Index; aBMD, areal bone mineral density; vBMD, volumetric BMD; BMI, body mass index; mSASSS, modified Stoke Ankylosing Spondylitis Spine Score; PLT, platelet count; WBC, white blood cell count.

A low BMD T-score was defined as a T-score < -1.0 measured with DXA at the lumbar spine (AP projection), total hip or femoral neck. Logistic regression with a forward conditional method was run with low T-score (yes/no) as the binary outcome and the variables that were significantly correlated with BMD as covariates. Significant covariates for low T-score were long time since onset of AS symptoms (B = 0.036, *P *= 0.014, odds ratio 1.727, 95% confidence interval 1.007 to 1.068), high BASMI (B = 0.301, *P *= 0.016, odds ratio 1.351, 95% confidence interval 1.057 to 1.068), low BMI (B = -0.162, *P *< 0.001, odds ratio 0.850, 95% confidence interval 0.781 to 0.925) and for women menopausal status (B = 0.869, *P *= 0.040, odds ratio 2.384, 95% confidence interval 1.041 to 5.461).

### Comparison of anteroposterior DXA and lateral DXA

Significantly more women had a lumbar spine BMD T-score ≤ -2.5 (age ≥ 50) or Z-score ≤ -2.0 (age < 50) when measured by lateral DXA (*n *= 23, 26%) compared with AP DXA (*n *= 14, 16%; *P *= < 0.001) (Table 2). T- and Z-scores for lateral spine DXA were not available for the men.

Lumbar spine BMD was significantly higher measured in the AP compared with the lateral projection (*P *< 0.001) (Table 1). The difference (AP minus lateral projection DXA BMD) increased with increasing mSASSS (r_S _= 0.389, *P *< 0.001), BASMI (r_S _= 0.296, *P *= 0.001), age (r_S _= 0.309, *P *= 0.001) and disease duration (r_S _= 0.268, *P *= 0.004) in men, but not in women.

**Figure 2:**
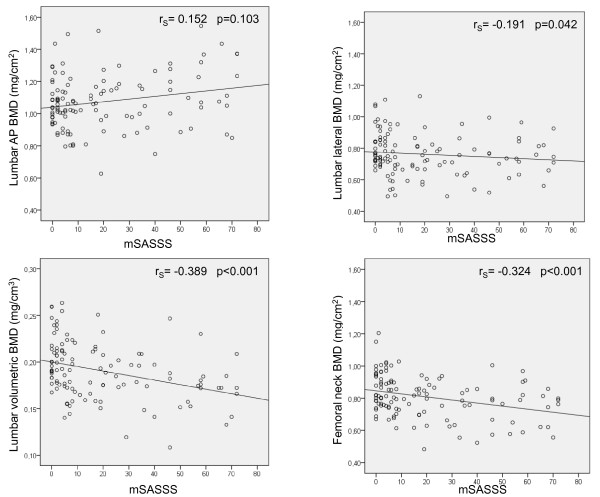
**Scatter plots of modified Stoke Ankylosing Spondylitis Spine Score (mSASSS) and BMD measured at different locations**. Spearman's correlation coefficients (r_S_) and *P*-values for the correlations are given.

Since men had significantly higher mSASSS than women the male subgroup was chosen to further study the effects of increasing ankylosis on BMD. Increasing mSASSS in men was significantly correlated with lower lumbar spine vBMD (r_S _= -0.389, *P *< 0.001), lower lateral spine BMD (r_S _= -0.191, *P *= 0.041), femoral neck BMD (r_S _= -0.324, *P *< 0.001) total hip BMD (r_S _= -0.201, *P *= 0.030) and total radial BMD (r_S _= -0.269, *P *= 0.004), but not with AP lumbar spine BMD (r_S _= 0.152, *P *= 0.103) (Figure 2). Higher BASMI in men also correlated significantly with lower BMD at all measurement sites, except for AP lumbar spine BMD, which tended to be higher, but was not significantly so.

## Discussion

The present study demonstrates that osteoporosis is common in Swedish patients with AS, but is often undiagnosed and thus untreated. BMD was below the expected range for age in 5% of patients under the age of 50 years. Osteoporosis, as defined by the WHO, was diagnosed in 21% of patients aged 50 years or older (in 30% of the women and 14% of the men). Most patients with osteoporosis were undiagnosed before the study. The prevalence of osteoporosis found in the present study is comparable with the prevalence in the Swedish general population aged 50 to 80 years, where 21.2% of women and 6.3% of men have been identified as having osteoporosis [25]. The results indicate that the increased risk of osteoporosis in AS compared with the general population is especially accentuated in male patients, which is in accordance with findings in another study [26].

In the current study osteoporosis and osteopenia in the lumbar spine were more common in women than in men in the age-group of 50 years or older when measured with AP lumbar DXA, and thus more women were diagnosed as osteoporotic. The prevalence of osteoporosis and osteopenia was equal among women and men at all other locations. The high prevalence of syndesmophytes in the men may have resulted in falsely elevated BMD causing an underestimation of male osteoporosis at the lumbar spine.

The results from the present study indicate that osteoporosis and osteopenia affect both the central and the peripheral skeleton. We found almost as many patients with BMD below the expected range for age or osteoporosis at the radius (*n *= 17, 8%) as in the lumbar spine (*n *= 24, 12%). Results from earlier studies indicate that osteoporosis in AS is predominantly confined to the central skeleton [27,28]. In one study no correlation was found between bone density at the forearm measured with peripheral quantitative computed tomography (pQCT) and DXA and quantitative CT (QCT) measurements of BMD at the lumbar spine or hip [29]. Quantitative ultrasound studies of the heel in patients with AS have inconsistent findings, with normal results in one study, and signs of peripheral osteoporosis in another [30,31]. The theory that osteoporosis is a general process affecting the whole skeleton in AS was supported by a study of bone biopsies from the iliac crest, showing trabecular thinning and low trabecular peripheral bone volume strongly correlated with lumbar spine BMD measured using QCT [32].

There is uncertainty about how to treat osteoporosis in patients with AS. Bisphosphonates have been studied in respect to their effect on disease activity in AS, but their effects on fractures, BMD and the new bone formation in AS needs to be further investigated. Pamidronate has been reported to hamper disease activity in AS [33,34]. In a recent placebo-controlled study of alendronate 70 mg weekly, no improvement of AS symptoms or disease activity was found [35].

In the current study we found that low BMD was associated with older age, longstanding disease, syndesmophyte formation, impaired back mobility, history of coxitis, use of glucocorticoids and laboratory signs of inflammation. Menopause was a strong risk factor for women. No connection was found between low BMD and the disease indices BASDAI, BASFI, BAS-G or ASDAS. BASMI and ASDAS were associated with inflammatory parameters (ESR, CRP), but BASDAI, BASFI and BAS-G were not.

The association between extensive syndesmophyte formation, restriction of spinal movement and osteoporosis has been demonstrated previously [26,29,36]. One study found significant correlation between low lumbar spine BMD and elevated ESR and CRP [37]. Two follow-up studies have shown that patients with AS and persistent inflammation, that is, with elevated ESR or CRP, developed significant decreases in BMD, whereas patients with low inflammatory activity did not [38,39].

In men, who had significantly higher mSASSS than women, increasing mSASSS and BASMI were significantly associated with decreasing vBMD and lateral BMD at the lumbar spine, along with lower BMD at the hip and radius, while AP lumbar BMD had a non-significant tendency to increase. Our interpretation of the results is that in comparison with AP spine BMD, lumbar spine vBMD and lateral lumbar spine BMD are less affected by the new bone formation in AS and hence are more adequate tools in assessing osteoporosis and osteopenia, especially in male patients in AS.

Lateral lumbar spine BMD is usually lower than AP BMD, because the lateral DXA scan measures only the trabecular-rich vertebral body, whereas the AP scan includes both the vertebral body and the posterior part of the vertebra, mainly consisting of dense cortical bone. AP scanning is also affected by artefacts due to to osteophytes, aortic calcifications and degenerative changes in the facet joints of elderly people and from hyperostosis in AS. The trabecular bone is more metabolically active than the cortical bone, therefore a decrease in BMD is first found in the trabecular bone. Consequently lateral lumbar spine BMD declines faster than AP BMD in early osteoporosis [40,41].

The current study demonstrates that lateral lumbar spine DXA is more sensitive than AP DXA in detecting osteoporosis and osteopenia in AS. The same results have been reported in two studies using lateral DXA of vertebra L3 in patients with AS [42,43]. Other studies have shown that lateral DXA is more sensitive than AP DXA in detecting osteopenia and osteoporosis in degenerative spinal disease [44]. In one study of 100 AS patients and 58 healthy controls assessed with both AP and lateral lumbar DXA using a scanner similar to the one used in the current study, the authors reported that lumbar spine BMD was significantly lower in AS patients compared with healthy controls when measured by lateral projection DXA, but not when measured by AP DXA [45]. However, to apply lateral DXA in clinical practice, reference intervals based on measurements on large populations of healthy men and women are required. Most likely, new threshold values for definition of osteoporosis have to be defined to avoid overestimation of osteoporosis with lateral DXA. The current WHO definition of osteoporosis is based on the PA projection and according to the Official Positions of the International Society for Clinical Densitometry 2007, the lateral spine should not be used for diagnosis of osteoporosis, but it may have a role in monitoring [23].

## Conclusions

Osteoporosis and osteopenia are common in Swedish patients with AS and affected half of our study population. Low BMD was found in both the central and the peripheral skeleton. Osteoporosis was often undiagnosed and untreated, particularly in male patients with AS. Older age and high disease burden, that is, long disease duration, impaired back mobility, syndesmophyte formation and elevated inflammatory parameters, indicated increased risk of osteoporosis. Lateral and vBMD at the lumbar spine were less affected by progressive ankylosis in AS compared with AP BMD. In addition, lateral DXA was more sensitive in detecting osteoporosis and osteopenia than AP DXA. We suggest that lateral lumbar spine DXA with calculation of vBMD may become valuable tools in the diagnosis and follow-up of osteoporosis in AS, but validation of the methods in larger reference populations is needed.

## Abbreviations

aBMD: areal bone mineral density; AP: anteroposterior; AS: ankylosing spondylitis; ASDAS: Ankylosing Spondylitis Disease Activity Score; BASDAI: Bath Ankylosing Spondylitis Disease Activity Index; BASFI: Bath Ankylosing Spondylitis Functional Index; BAS-G: Bath Ankylosing Spondylitis Patient Global Score; BASMI: Bath Ankylosing Spondylitis Metrology Index; BMI: body mass index; CRP: C-reactive protein; DMARD: disease modifying anti-rheumatic drug; DXA: dual-energy x-ray absorptiometry; ESR: erythrocyte sedimentation rate; FRAX: fracture risk assessment tool; GC: glucocorticoid; HRT: hormone replacement therapy; LTPAI: Leisure Time Physical Activity Instrument; mSASSS: modified Stoke Ankylosing Spondylitis Spine Score; NSAID: non-steroidal anti-inflammatory drug; PAHWI: Physical Activity at Home and Work Instrument; PLT: platelet count; TNF: tumour necrosis factor; vBMD: volumetric BMD; WBC: white blood cell count; WHO: World Health Organization.

## Competing interests

The authors declare that they have no competing interests.

## Authors' contributions

EK participated in the design of the study, examined the patients, performed statistical analyses and drafted the manuscript. HC and HF supervised the study, and were responsible for study design and interpretation of data. HF contributed to statistical analyses. ML and DM were responsible for the bone mineral density measurements. MG and JG were responsible for assessing the spinal radiographs. EH and MH were responsible for including the patients from Alingsås and Borås. All authors have critically reviewed and approved the final manuscript.
